# Postinjury Inhibition of miR-181a Promotes Restoration of Hippocampal CA1 Neurons after Transient Forebrain Ischemia in Rats

**DOI:** 10.1523/ENEURO.0002-19.2019

**Published:** 2019-08-29

**Authors:** Brian B. Griffiths, Yi-Bing Ouyang, Lijun Xu, Xiaoyun Sun, Rona G. Giffard, Creed M. Stary

**Affiliations:** Department of Anesthesiology, Perioperative and Pain Medicine, Stanford University School of Medicine, Stanford, CA 94305-5117

**Keywords:** astrocytes, forebrain ischemia, hippocampus, microRNA, neurogenesis

## Abstract

The cellular and molecular mechanisms regulating postinjury neurogenesis in the adult hippocampus remain undefined. We have previously demonstrated that preinjury treatment with anti-microRNA (miR)-181a preserved neurons and prevented astrocyte dysfunction in the hippocampal cornu ammonis-1 (CA1) following transient forebrain ischemia. In the present study, we assessed postinjury treatment with anti-miR-181a on recovery of CA1 neurons following transient forebrain ischemia in rats. Stereotactic CA1 injection of miR-181a antagomir at either 2 h or 7 d postinjury resulted in improved restoration of CA1 measured at 28 d postinjury. Treatment with antagomir was associated with overexpression of the mir-181a target cell adhesion-associated, oncogene-related protein and enhanced expression of the neuroprogenitor cell marker doublecortin (DCX) in the CA1. Assessment of GFAP^+^ cell fate by Cre/Lox-mediated deletion demonstrated that some GFAP^+^ cells in CA1 exhibited *de novo* DCX expression in response to injury. *In vitro* experiments using primary neuronal stem cells confirmed that miR-181a inhibition augmented the expression of DCX and directed cellular differentiation toward a neuronal fate. These results suggest that miR-181a inhibition plays a central role in the restoration of CA1 neurons via augmentation of early latent neurogenic gene activation in neural progenitor cells, including some reactive astrocytes. Therapeutic interventions targeting this restorative process may represent a novel postinjury approach to improve clinical outcomes in survivors of forebrain ischemia.

## Significance Statement

Persistent cognitive impairment is a major source of decreased quality of life for survivors of cardiac arrest, with impaired memory cited as the most severe long-term deficit (Moulaert et al., 2009). Interruptions in cerebral blood flow result in delayed death of hippocampal cornu ammonis-1 (CA1) neurons (Horn and Schlote, 1992). Pharmaceutical therapies given during this 2 week therapeutic window could drastically reduce the cognitive deficits experienced by survivors of forebrain ischemia. However, interventions that have attempted to directly target neurons after forebrain ischemia have so far failed to translate to effective therapies. Therefore, there is an urgent need for novel treatments that either provide protection against loss or hasten functional recovery of CA1 neurons.

## Introduction

In response to forebrain ischemia, the adult brain attempts to repair damage by producing new neurons not only in known neurogenic regions, but also in areas where neurogenesis does not normally occur, including the striatum (Magnusson et al., 2014) and hippocampal cornu ammonis-1 (CA1; Schmidt and Reymann, 2002; Bendel et al., 2005; Salazar-Colocho et al., 2008). In young adult rodent models of forebrain ischemia, neuronal recovery of CA1 is a well established phenomenon (Kirino, 1982); however, cell division is not a significant factor in observed neuronal recovery (Sugawara et al., 2000). In adult humans, delayed neuronal death is similarly a principle factor that contributes to impaired cognitive function following global cerebral ischemia (Horn and Schlote, 1992). However *de novo* neuronal proliferation and numbers of resident neuroprogenitor cells in the hippocampus drastically decrease after normal childhood brain development (Boldrini et al., 2018). Therefore, proliferative neurogenesis from resident neuroprogenitor cells cannot fully explain postinjury restoration of CA1 neurons after forebrain ischemia in adults.

Astrocytes, specialized glia that regulate neuronal homeostasis in health and injury, have been demonstrated to play a central role in neurogenesis in the adult brain (Cassé et al., 2018). We and others have provided increasing evidence of a protective role for astrocytes against ischemic injury in the adult (Ouyang et al., 2014; Stary and Giffard, 2015; Li and Stary, 2016). Astrocyte protection of CA1 neurons can be augmented by manipulation of endogenous expression of microRNAs (miRs), a class of small (19–22 nt) noncoding RNAs that regulate gene expression primarily at the post-transcriptional level. In particular, miR-181a is highly expressed in the adult brain after injury, and we have previously demonstrated that both pretreatment and post-treatment with miR-181a antagomir improves protection and recovery after focal cerebral ischemia (Ouyang et al., 2012b; Xu et al., 2015). In the setting of forebrain ischemia, we have previously demonstrated that miR-181a antagomir preinjury treatment preserves astrocyte function and protects CA1 neurons from delayed cell death (Moon et al., 2013). miR-181a has been previously established to play a role in embryonic stem cell development of neurons through direct inhibition of cell adhesion-associated, oncogene-related (CDON) protein expression, a critical regulator of embryonic neurogenesis (Gibert et al., 2014). Therefore, in the present study we did the following: (1) explored *in vivo* the effect of postinjury anti-miR-181a treatment on the restoration of hippocampal CA1 neurons after forebrain ischemia, and the role astrocytes may play in this process; and, (2) assessed the effect of miR-181a in determining the neurogenic fate of stem cells *in vitro.*

## Materials and Methods

### Forebrain ischemia

All experimental protocols using animals were performed according to protocols approved by the Stanford University Animal Care and Use Committee and in accordance with the National Institutes of Health *Guide for the Care and Use of Laboratory Animals*. Forebrain ischemia was induced via two-vessel occlusion plus hypotension in adult (3 months of age; 300-400 × *g*), male Sprague Dawley rats (Charles River Laboratories), a model that reliably results in select destruction of CA1 neurons as described previously (Ouyang et al., 2007, 2012b). Briefly, hypotension (mean arterial pressure, <40 mmHg) was induced by removing blood into heparinized sterile tubing during continuous femoral arterial blood pressure monitoring (Puritan Bennett). Carotid arteries were clamped bilaterally simultaneously for 10 min. Rectal temperature (37 ± 0.5°C) was controlled by a homeothermic blanket (Harvard Apparatus). Respiratory rate, heart rate, and pulse oximetry were monitored with a small animal oximeter (STARR Life Sciences). After 10 min, the clamps were removed and shed blood was reinfused.

### *In vivo* treatments

Animals were randomly assigned to treatment group by coin flip to receive either stereotactic intraparenchymal injection with 5′6-FAM fluorescently labeled miR-181a-5p antagomir (3 pmol/g; Thermo Fisher Scientific) mixed 1:3 with the cationic lipid DOTAP (catalog #11202375001, Roche Applied Science); or 5′6-FAM fluorescently labeled mismatch control (MM-con) treatment. The stereotactic injections were administered at either 2 h or 7 d after injury, and miR-181a antagomir or MM-con was stereotactically infused in the stratum moleculare of the CA1, allowing miR-181a or MM-con to perfuse into the CA1, CA3, and DG, as previously described (Ouyang et al., 2007, 2012b). See Fig. 3*A* for a visual representation. Animals were killed at 7, 14, 21, 28, 70, or 91 d after injury, and brains were fixed for histologic assessment.

### Fluorescent immunohistochemistry

Ischemic or sham-operated rats were deeply anesthetized and transcardially perfused with cold 0.9% saline, followed by 4% paraformaldehyde (PFA) in PBS, pH 7.4. Immunohistochemistry was performed as described previously (Xu et al., 2015). Briefly, brains were kept refrigerated in 4% PFA in PBS, pH 7.4, for at least 3 d and then processed into 50 μm sections with a vibratome (VT1000S, Leica Microsystems). Brain sections were blocked in serum overnight, incubated in primary antibodies (Table 1) overnight and then incubated in secondary antibodies overnight (Table 2). Immunofluorescent images of the marker for mature neuronal somas NeuN, the neuroprogenitor marker doublecortin (DCX), the astrocyte markers glial fibrillary acidic protein (GFAP) and CDON (expressed in neuronal precursors) were acquired in hippocampal sections using an upright Zeiss Axio lmager M2 Multichannel Fluorescent Microscope. CA1 NeuN^+^ cell populations were adjusted for total cell count using the nuclear stain 4′,6′-diamidino-2-phenylindole dihydrochloride (DAPI; catalog #10236276001, Sigma-Aldrich) and quantitated using 3D optical sectioning in the *z*-axis by structured illumination with an Apotome 2.0 using StereoInvestigator software. The images presented are as close to representative as possible, although the conversion of 3D images to 2D may obscure some overlapping cells. Cellular colocalization was determined in 3D image stacks using Neurolucida version 17 software. For DCX and CDON staining analysis in the CA1 pyramidal layer, image stacks were analyzed using ImageJ by creating a threshold and measuring the mean illumination for each image section, collapsing each set of measurements to a single value for each animal.

**Table 1.** Primary antibodies used for fluorescent immunohistochemistry

Protein	Host	Manufacturer	Dilution
NeuN	Mouse	EMD Millipore, catalog #MAB377; RRID:AB_2298772	1:500
NeuN	Rabbit	Abcam, catalog #ab177487; RRID:AB_2532109	1:500
GFAP	Mouse	Abcam, catalog #ab10062; RRID:AB_296804	1:500
GFAP	Rabbit	Abcam, catalog #ab5804; RRID:AB_2109645	1:1000
GFAP	Chicken	Abcam, catalog #ab4674; RRID:AB_304558	1:500
DCX	Rabbit	Abcam, catalog #ab18723; RRID:AB_732011	1:1000
CDON	Mouse	Santa Cruz Biotechnology, catalog #sc-377232	1:500
MAP2	Chicken	Abcam, catalog #ab5392; RRID:AB_2138153	1:500

**Table 2.** Secondary antibodies used for fluorescent immunohistochemistry

Fluorophore	Host	Manufacturer
Alexa Fluor 488	Donkey anti-rabbit IgG	Thermo Fisher Scientific, catalog #A21206 RRID:AB_2535792
Alexa Fluor 488	Donkey anti-goat IgG	Thermo Fisher Scientific, catalog #A11055 RRID:AB_2534102
Alexa Fluor 488	Goat anti-chicken IgG	Thermo Fisher Scientific, catalog #A32931 RRID:AB_2340375
Alexa Fluor 594	Donkey anti-goat IgG	Thermo Fisher Scientific, catalog #A11058 RRID:AB_2534105
Alexa Fluor 594	Donkey anti-mouse IgG	Thermo Fisher Scientific, catalog #A21203 RRID:AB_141633
Alexa Fluor 594	Donkey anti-sheep IgG	Thermo Fisher Scientific, catalog #A11016 RRID:AB_2534083
Alexa Fluor 594	Donkey anti-rabbit IgG	Thermo Fisher Scientific, catalog #SA5-1005 RRID:AB_2556620

### *In vitro* treatments

Neural precursor cells were isolated postnatally from newborn mice at 24 h. The brains were removed, freed of meninges, and diced with a sterile razor blade in dissociation buffer (DMEM; catalog #D5796, Sigma-Aldrich) containing 2.5 U/ml papain (catalog #1495005, Sigma-Aldrich), 250 U/ml DNase I (catalog #LS006330, Worthington Biochem), and 1 U/ml Dispase II (catalog #04942078001, Roche Diagnostics). After a 1 h incubation at 37°, the cells were washed three times with DMEM supplemented with 10% fetal bovine serum (FBS; HyClone FBS; Cat # SH3007103, Thermo Fisher Scientific). The cells were then resuspended in growth medium, Gibco Neurobasal A (catalog #21103049, Thermo Fisher Scientific), with 2 mM l-glutamine, 100 U/ml Gibco penicillin-streptomycin (catalog #15140122, Thermo Fisher Scientific), B_27_ without vitamin A (catalog #A3353501, Thermo Fisher Scientific), 20 ng/ml fibroblast growth factor-2 (catalog #100-18B, Peprotech), and 20 ng/ml epidermal growth factor (catalog #AF-100-15, Peprotech); and plated at a density of 1 brain per six-well plate. Neural precursor cells proliferated and started to form neurospheres in 2–3 d, the plates were shaken every day to prevent attachment. Neural precursor cells were induced to differentiate by plating dissociated neurospheres in laminin-coated 24-well plates, (the plates were coated with 10 μg/well Gibco laminin (catalog #23017015, Thermo Fisher Scientific) in distilled H_2_O (dH_2_O) for 2–3 h and rinsed twice with dH_2_O. The floating neurospheres were collected after passage through a 70 μm cell strainer (catalog #087712, Thermo Fisher Scientific) by centrifugation at 400 × *g* for 5 min at 4°C. The cell pellet was gently triturated with a 200 μl pipette tip 15–20 times, resuspended in a small volume of differentiation medium, Neurobasal A, B27, 1% FBS, 1 ng/ml fibroblast growth factor-2, 10 ng/ml brain-derived neurotrophic factor (catalog #450-02, Peprotech), and 10 ng/ml neurotrophic factor-3 (catalog #450-03; Peprotech). The cells were plated onto laminin-coated 24-well plates at a density of 200,000 cells/ml. One-half of the differentiation medium was changed every 2–3 d. Cells were grown in 21% O_2_/5% CO_2_ and were allowed to differentiate for different times dependent on the experimental design as detailed previously (Sun et al., 2015). Transfection started on day 1 after cells were grown in differentiation medium. To transfect cells, 50 pmol miR-181a-5p mimic or inhibitor was used with FuGENE HD Reagent (catalog #E2311, Promega) every 3 d, and the transfection reagent was washed out after overnight incubation. All cell culture experiments were repeated in triplicate.

### Immunocytochemistry

Fluorescence immunocytochemistry was assessed in cell cultures grown in 24-well plates. The cultures were washed with PBS and then fixed in 4% PFA for 30 min at room temperature. The cells were then washed three times with PBS, and nonspecific binding was blocked with 5% normal horse serum and 0.1% Triton X-100 in PBS for 1 h. The cells were subsequently incubated with primary antibodies diluted in blocking buffer overnight at 4°C (Table 1). Cells were washed three times in PBS plus 0.1% Triton X-100 buffer and subsequently incubated with the appropriate secondary antibodies (Table 2). Cell nuclei were counterstained with DAPI (0.5 μg/ml; Sigma-Aldrich). To eliminate observer selection bias, immunofluorescent images (nine per well) were acquired using an automated epifluorescence microscope (LS720, Etaluma; Lumaview software version 17.11, Etaluma). Fluorescence intensity was then quantified using Fiji (version 1.51s) by an observer blinded to conditions.

### Fate mapping DCX/GFAP Cre-Lox system

We designed a DCX/GFAP plasmid to track *de novo* DCX expression in GFAP-producing cells. The plasmid contained a DCX promoter linked to both CRE and mCherry expression, and a floxed GFAP promoter-EGFP sequence. In the absence of DCX expression, EGFP (green) is expressed from a GFAP promoter in transfected astrocytes, which express GFAP. On activation of the DCX promoter, Cre and mCherry (red) are coexpressed. Cre removes the floxed GFAP promoter-EGFP sequence and EGFP expression is terminated, while DCX^+^ cells express mCherry. Cells expressing only EGFP lack DCX expression, but any EGFP^+^/mCherry^+^ cells indicate *de novo* expression of DCX in a GFAP^+^ cell. DCX (Voloboueva et al., 2010) and GFAP (Xu et al., 2010) promoters were obtained from prior clones (listed in Table 3). The plasmid was sterotactically injected 24 h before ischemia, and animals were killed 7 d after ischemia and treated as detailed in the immunohistochemistry methods above.

**Table 3.** Primers for cloning fate-mapping plasmid promoter sequences

Promoter	PCR Primers
DCX	CTCGAGATATTCTTATCGCCGCACATC GGATCCTTGGTGGAACCACAGCAACCTGA
GFAP	GCTAGCTTGAGCCGGGCAGTGT AAGCTTACGTAGCGTGGTTTAC

### Statistical analysis

The numbers of animals per group are indicated in the figure legends. Data are reported as the mean ± SEM. For cell culture experiments, technical replicates (at least four wells) were averaged and treated as a single sample, and all experiments were repeated in triplicate (three separate dissections). All statistical analyses were performed on SPSS (version 22, IMB; RRID:SCR_002865). Statistical difference was determined using Student’s *t* test for comparison of two groups with Cohen’s d as a measure of effect size, or ANOVA for experiments with more than two groups with partial η^2^ as a measure of effect size with Tukey’s *post hoc* test for group comparisons. In all analyses, *p* < 0.05 was considered statistically significant.

## Results

### Loss and restoration of hippocampal CA1 neurons following forebrain ischemia

**Figure 1.**
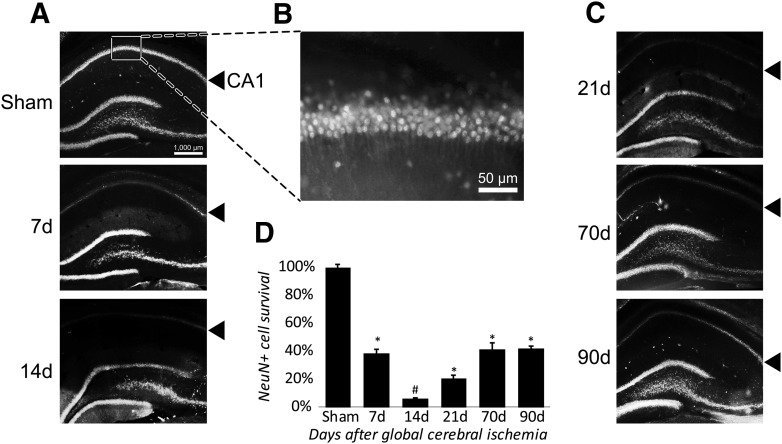
CA1 neuronal loss and recovery following forebrain ischemia. ***A***, Forebrain ischemia results in delayed cell death of mature (NeuN^+^) neurons in hippocampal CA1 (denoted by solid arrowhead). ***B***, Magnified view of CA1 neurons in sham animals. ***C***, Gradual and partial postinjury recovery of NeuN^+^ neurons in CA1. ***D***, Quantitative measurement of postinjury loss and recovery of CA1 neurons. *N* = 3-6/group,*p < 0.05 compared with sham, #*p* < 0.05 compared with every group. Error bars are mean ± SEM.

An established characteristic of the injury model used in the present study is delayed, extensive loss of hippocampal CA1 pyramidal neurons (Pulsinelli et al., 1982). Immunostaining for NeuN, a marker for mature neurons, confirmed the expected loss of CA1 neurons after forebrain ischemia (Fig. 1*A*; *F*_(5,15)_ = 315.24, *p* < 0.001, partial η^2^ = 0.99). *Post hoc* analysis indicated that neuronal loss had occurred by 7 d after ischemia with the number of CA1 neurons dropping to ∼38% compared with sham controls. By 14 d after ischemia, the CA1 neuronal population was almost completely abolished with only ∼6% of NeuN^+^ cells remaining compared with sham animals (Fig. 1*B*). Significant (*p* < 0.01) recovery of mature neurons was observed by 21 d (Fig. 1*C*) relative to the 14 d nadir, and continued to increase to a plateau of ∼41% of the original number by 70 d, after which the number of NeuN^+^ neurons remained mostly unchanged by 90 d postinjury (Fig. 1*B*,*C*).

### Reactive astrocytes were induced after forebrain ischemia

**Figure 2.**
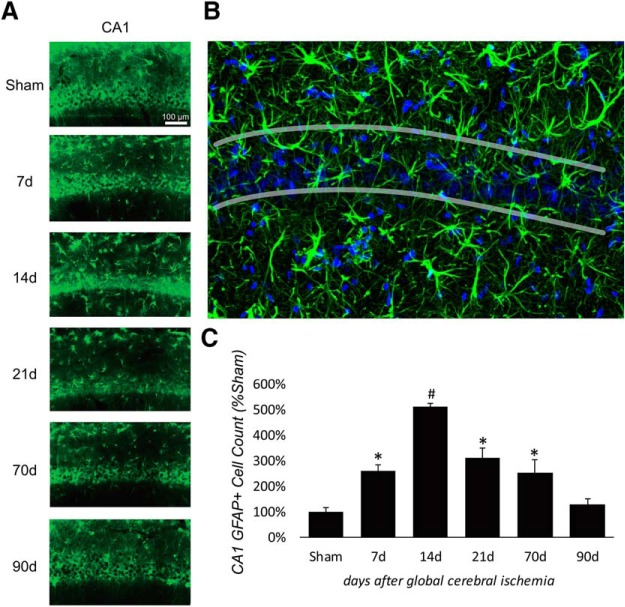
Hippocampal increased GFAP expression in the CA1 pyramidal layer following forebrain ischemia. ***A***, Fluorescent labeling of GFAP after forebrain ischemia in the hippocampus (50× magnification). ***B***, Enlarged (200×) example of CA1 pyramidal layer at 14 d after forebrain ischemia. ***C***, Postinjury quantification of GFAP^+^ cells in CA1. *N* = 3-6/group, *p < 0.05 compared with sham, #*p* < 0.05 compared with every group. Error bars are mean ± SEM.

In conjunction with the reappearance of mature neurons after forebrain ischemia, hippocampal GFAP^+^ astrocytes were quantitated via immunohistochemical labeling. In sham control animals, GFAP^+^ cells in CA1 were not localized to the pyramidal neuronal layer. Following forebrain ischemia, we observed a significant increase of GFAP^+^ cells in the CA1 pyramidal layer compared with sham controls by 7 d postinjury, which was sustained until 70 d postinjury (*F*_(5,33)_ = 42.55, *p* = < 0.001, partial η^2^ = 0.87; Fig. 2*A*). *Post hoc* analysis revealed that the number of GFAP^+^ cells peaked at 14 d postinjury, 7 d before when CA1 first demonstrated observable recovery of mature neurons (Fig. 2*B*). By 91 d, the total number of GFAP^+^ cells in hippocampus had largely returned to the preinjury baseline (Fig. 2*C*). To examine whether postinjury miR-181a inhibition enhanced the recovery process, we treated animals with antagomir either immediately (2 h) after injury or 7 d after injury, time points that precede both maximum cell death (14 d; Fig. 1*D*) and the maximum number of reactive astrocytes in the CA1 (14 d; Fig. 2*B*).

### Enhanced recovery of hippocampal CA1 neurons by miR-181a antagomir

**Figure 3.**
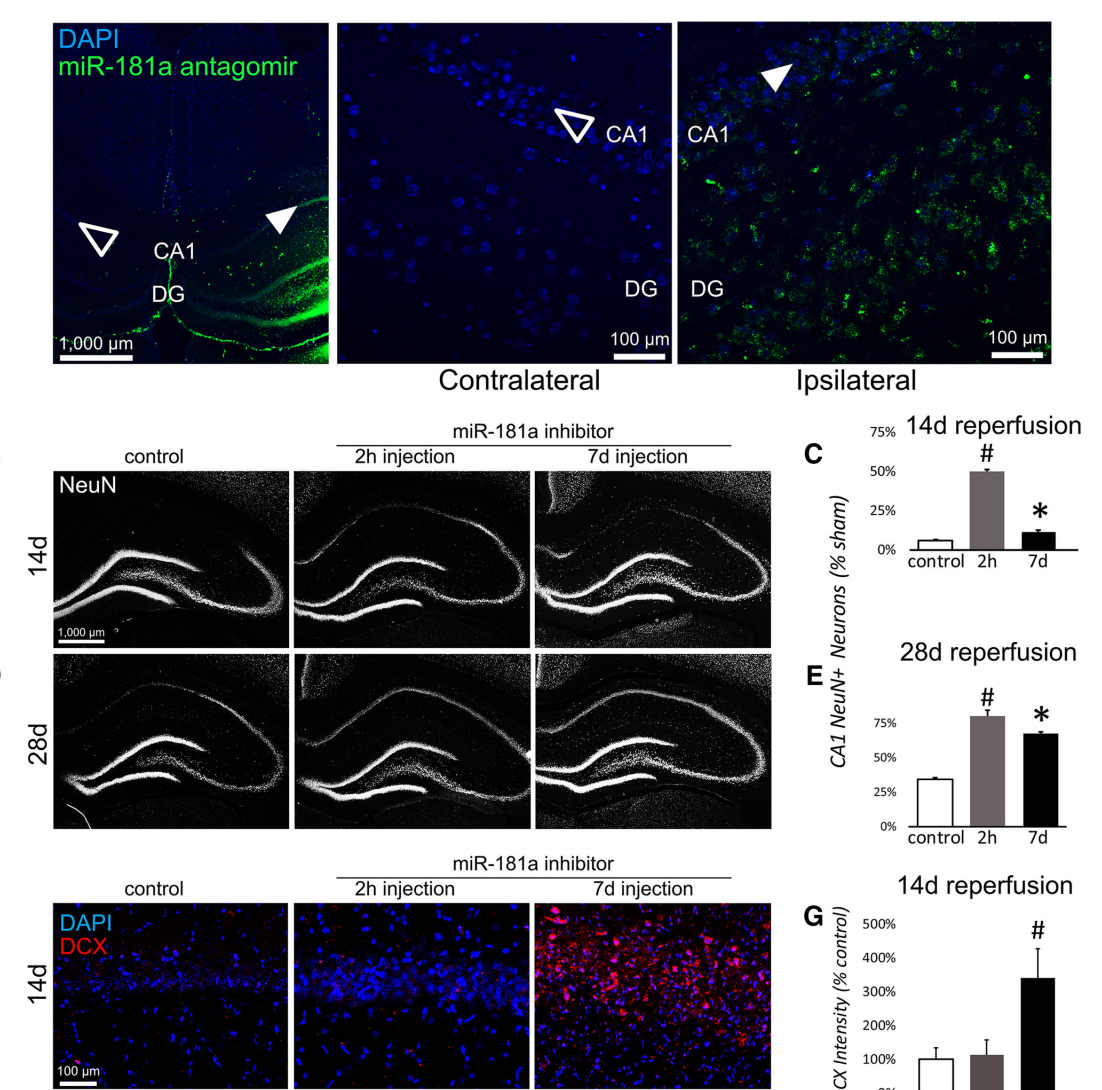
Effect of postinjury miR-181a antagomir treatment on CA1 NeuN and DCX precursor expression and long-term CA1 neuronal recovery following forebrain ischemia. ***A***, Fluorescently labeled (green, 6-FAM) miR-181a antagomir is detected in ipsilateral hippocampus (solid arrowhead), but not in contralaterally injected hippocampus (empty arrowhead). Magnified views of contralateral and ipsilateral CA1 following injection of 6-FAM-labeled miR-181a antagomir. ***B***, ***C***, Examples (***B***) and quantification (***C***) of ipsilateral hippocampal NeuN fluorescent labeling 14 d after forebrain ischemia in postinjury MM-con-treated and antagomir-treated animals. ***D***, ***E***, Examples (***D***) and quantification (***E***) of ipsilateral hippocampal NeuN fluorescent labeling at 28 d postinjury in MM-con-treated and antagomir-treated animals. ***F***, ***G***, Examples (***F***) and quantification (***G***) of ipsilateral CA1 DCX^+^ cells 14 d after forebrain ischemia with MM-con or antagomir treatment 2 h or 7 d after forebrain ischemia. *N* = 5-6 for each condition. *p < 0.05 compared with sham, #*p* < 0.05 compared with every group. Error bars are mean ± SEM.

Stereotactic injection of fluorescently labeled miR-181a antagomir exhibited robust transduction throughout CA1 and DG in the ipsilateral hippocampus (Fig. 3*A*) but was completely absent in the contralateral hippocampus. For all experiments, only the ipsilateral side of the hippocampus that received either the MM-con of miR-181a inhibitor treatment was analyzed. There were no differences between 2 h and 7 d MM-con-treated animals. MiR-181a antagomir significantly increased the survival of mature neurons in CA1 14 d after ischemia when injected 2 h or 7 d postinjury (Fig. 3*B*,*C*; *F*_(2,20)_ = 124.76, *p* < 0.001, partial η^2^ = 0.93). Interestingly, both the 2 h and 7 d injection exhibited enhanced recovery of CA1 neurons relative to controls when assessed 28 d after ischemia, though the animals receiving the 2 h injection demonstrated slightly greater recovery than those receiving the 7 d injection (Fig. 3*D*,*E*; *F*_(2,22)_ = 456.36, *p* < 0.001, partial η^2^ = 0.98). Further inspection of the CA1 revealed that animals injected with miR-181a antagomir at 7 d after ischemia had significantly greater levels of DCX fluorescence in the CA1 pyramidal layer than those treated at 2 h (Fig. 3*F*,*G*; *t*_(7)_ = 2.50, *p* = 0.041, Cohen’s d = 3.32).

### Promotion of neurogenesis by miR-181a antagomir through CDON overexpression

**Figure 4.**
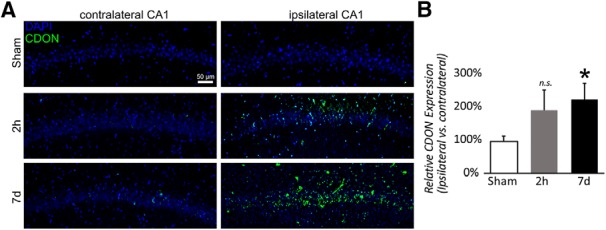
Postinjury CDON expression in CA1 following forebrain ischemia. ***A***, Immunofluorescent labeling of CDON expression (green) and all nuclei (DAPI) in CA1 14 d after forebrain ischemia with MM control treatment or 2 h or 7 d postinjury antagomir treatment. ***B***, Quantification of CDON in CA1 14 d after forebrain ischemia with the three treatment groups. *N* = 4 for each condition, *p < 0.05 difference versus sham. Error bars are mean ± SEM. n.s. = not significant.

The embryonic regulator of neurogenesis, CDON, has been previously identified as a target of miR-181a (Gibert et al., 2014). In the adult rats used in the present study, CDON expression was largely undetectable 14 d following sham surgery (data not shown) and was only minimally detected after forebrain ischemia in treated MM-con animals (Fig 4*A*). Treatment with miR-181a antagomir 7 d postinjury resulted in a significant increase in hippocampal CDON expression in the CA1 pyramidal layer (Fig. 4*B*; *t*_(7)_ = −2.45, *p* = 0.040, Cohen’s d = −1.61). MiR-181a antagomir treatment at 2 d postinjury demonstrated a similar trend, though it failed to reach significance (*p* = 0.161).

### Coexpression of DCX with GFAP after forebrain ischemia

**Figure 5.**
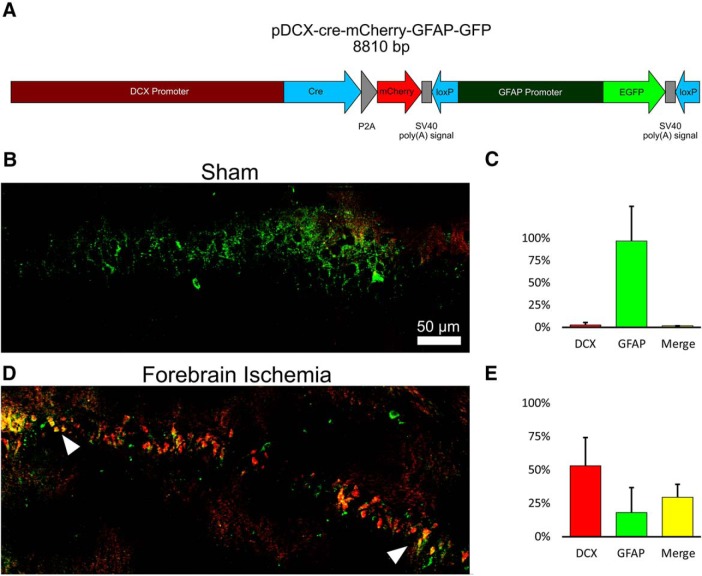
Fate-mapping postinjury DCX expression following forebrain ischemia. ***A***, Diagram and map of Cre-Lox GFAP/DCX fate-mapping plasmid. A floxed GFAP promoter EGFP (green) reporter and a DCX promoter controlling Cre and mCherry (red) reporter are included on the same plasmid. In the absence of DCX activity, astrocytes fluoresce green. With DCX activation, mCherry is expressed and EGFP expression is simultaneously terminated. ***B***, ***C***, Sham animals display very little new EGFP expression in CA1 after transfection, and no mCherry. ***D***, ***E***, Following forebrain ischemia (7 d), colocalization of EGFP/c-Tomato is evident in CA1, suggesting *de novo* expression of DCX in GFAP-expressing cells. The graphs indicate relative expression of DCX^+^ only, GFAP^+^ only, and DCX/GFAP^+^ double-positive cells. Error bars are mean ± SEM.

We see an increase in reactive astrocytes localized to the CA1 pyramidal cell layer at 14 d, and prior observations in adult animals have described *de novo* conversion of astrocytes to neuronal precursors cells following injury (Buffo et al., 2008; Gabel et al., 2016). We investigated whether this phenomenon could also account for CA1 neuronal recovery following forebrain ischemia. Immature neurons have been previously observed to coexpress GFAP in adult neurogenesis (Garcia et al., 2004), while mature astrocytes have been demonstrated to have the capacity to dedifferentiate and express DCX (Verwer et al., 2007). To differentiate between these two possible origins for DCX^+^ cells, we constructed a DCX/GFAP Cre-Lox fate-mapping plasmid (Fig. 5*A*). The 24 h following stereotactic injection of the plasmid sham animals displayed only EGFP (green) fluorescence in CA1 pyramidal layer (Fig. 5*B*,*C*), indicating successful transduction and reporting in astrocytes, but a lack of DCX activity in these GFAP^+^ cells. In contrast, forebrain ischemia after plasmid treatment resulted in robust expression of mCherry (red), indicating activation of DCX transcription, with substantial coexpression with EGFP (Fig. 5*D*,*E*). These observations suggest that at least a subset of DCX^+^ cells represented astrocytes that began to express DCX *de novo* in response to injury.

### miR-181a inhibition promotes neurogenesis in neural stem cells

**Figure 6.**
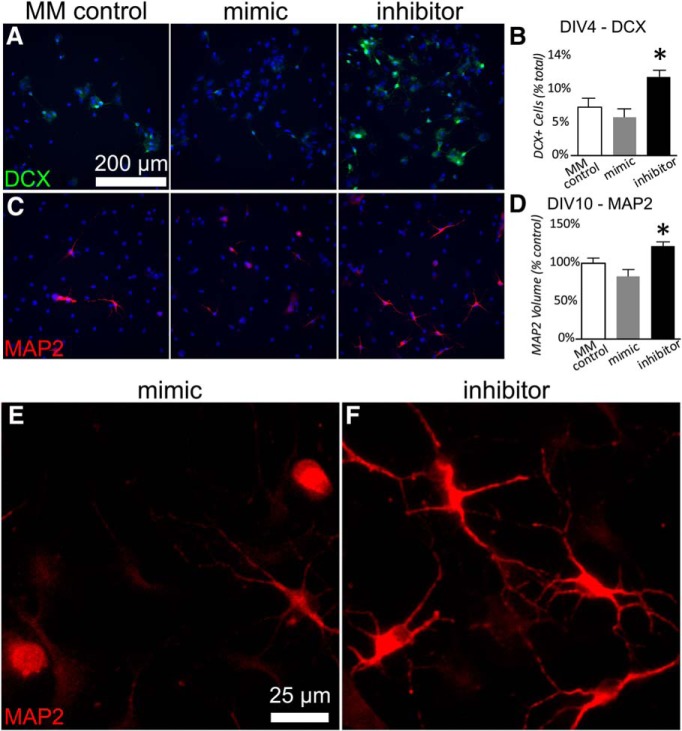
Effect of miR-181a in neural stem cell differentiation. ***A***, ***B***, Examples (***A***) and quantification (***B***) of DCX expression at DIV4 in neural stem cells treated with miR-181a inhibitor, mimic, or MM control. ***C***, ***B***, Examples (***C***) and quantification (***D***) of MAP2 expression at DIV10 in neuronal stem cells treated with miR-181a inhibitor, mimic, or MM control. Ten images were averaged for each sample well, then a grand average was generated for each condition. Experiments were repeated three times. The reported means represent the intracondition averages for the three experiments, *N* = 3 for each condition. Error bars are sample mean ± SEM. *p < 0.05 versus all other groups. ***E***, ***F***, Enlarged (400×) images of MAP2^+^ cells treated with miR-181a mimic (***E***) or inhibitor (***F***) reflecting qualitative differences in neuronal morphology between treatments.

To test the hypothesis that miR-181a contributes to the regulation of cellular differentiation, we altered miR-181a levels in growing neural stem cell cultures. By day *in vitro* 4 (DIV4), cells with suppressed miR-181a expression due to treatment with inhibitor demonstrated a marked increase in the number of DCX^+^ cells compared with cells treated with miR-181a mimic or controls (Fig. 6*A*,*B*; *F*_(2,26)_ = 7.54, *p* = 0.003, partial η^2^ = 0.37). Consistent with early elevations in DCX, cells assessed later at DIV10 demonstrated significantly greater expression of the mature neuronal marker MAP2 (microtubule associated protein 2) if miR-181a was suppressed with inhibitor compared with cells treated with mimic (Fig. 6*C*,*D*; *F*_(2,27)_ = 8.20, *p* = 0.002, partial η^2^ = 0.38). Qualitatively, immunocytochemical MAP2 staining also revealed morphologic differences in the mature neuronal population when miR-181a levels were altered: miR-181a inhibitor treatment tended to generate larger neurons with longer processes, versus treatment with mimic (Fig. 6*E*,*F*). The numbers of cells expressing the mature astrocyte marker GFAP were unaffected by miR-181a inhibitor or mimic (data not shown).

## Discussion

In the present study, we report that postinjury treatment with miR-181a antagomir at 2 h or 7 d increased the hippocampal CA1 neuronal count 28 d after forebrain ischemia. One difference between the two treatments was that 2 h postinjury treatment might be considered neuroprotective and restorative, while 7 d postinjury treatment was only restorative. Within the window of postinjury neuroinflammation, 2 h postinjury treatment likely prevented some of the initial loss of CA1 neurons, since CA1 neuronal cell counts did not fall as far when assessed on day 14, the nadir for CA1 neuronal loss. miR-181a inhibition has been shown to be protective in several other brain injury models, such as focal ischemia (Ouyang et al., 2012a; Xu et al., 2015; Stary et al., 2017), epilepsy (Ren et al., 2016), and Parkinson’s disease (Hegarty et al., 2018). Notably, miR-181a inhibition has been demonstrated to decrease inflammation (Xie et al., 2013a), and miR-181a is expressed early during the inflammatory response, but at lower levels in the chronic postinjury phase (Xie et al., 2013b). At 7 d postinjury, any acute inflammation from reperfusion injury would have subsided when CA1 neuronal loss already approached 60% (Fig. 1). Prevention from additional loss could not alone account for the observed difference at 28 d postinjury. Instead, our observations suggest that miR-181a inhibition instead augmented endogenous activation of local pluripotent cells and their development into NeuN^+^ neurons, at least in part originating from dedifferentiated astrocytes (Robel et al., 2011).

In the present study, we observed that 14 d postinjury coincided with both the peak of CA1 neuronal cell loss and the peak of GFAP expression (Figs. 1, 2). A similar time course has been observed in other brain injury models whereby latent activation of neurogenic pathways in astrocytes was observed as early as 1 week postinjury (Simon et al., 2011). Distinct from proliferative neurogenesis, several models have demonstrated the potential for astrocytes to dedifferentiate and reprogram into neurons in response to injury (Buffo et al., 2008; Robel et al., 2011; Magnusson et al., 2014). Prior studies have demonstrated that CA1 neurogenesis is not due to cell division, supported by observations of minimal BrdU labeling during the restorative phase in CA1 pyramidal layers (Sugawara et al., 2000). In the absence of proliferating or migrating neuroprogenitors as a source of new neurons in CA1, the activation of pre-existing localized cells remains a possibility. Neural progenitors are not prevalent in the CA1, but astrocytes are common (Zeisel et al., 2015). We observed DCX expression in GFAP^+^ cells in postinjury CA1 (Fig. 5), suggesting that astrocytes were not directly transdifferentiating, but instead that some may be dedifferentiating secondary to *de novo* expression of latent neuroprogenitor genes. Neurogenic properties of astrocytes appear to be dependent on local environmental factors, including Notch signaling (Imayoshi et al., 2008; Magnusson et al., 2014) and CDON expression. CDON is normally silenced through interactions with MeCP2 in astrocytes (Yasui et al., 2013); however, in the present study we observed a significant postinjury (14 d) increase in CDON expression (Fig. 4) in animals treated with miR-181a antagomir 7 d postinjury, but not in animals treated 2 h postinjury. It is not unreasonable to expect a residual effect on neurogenesis in CA1 with injection at 2 h postinjury, but this does not appear to be the primary mechanism.

Recent reviews have focused on specialized astrocytes that function as neural stem cells in the adult brain (Götz et al., 2015; Magnusson and Frisén, 2016; Platel and Bordey, 2016). After distal permanent middle cerebral artery occlusion surgery, self-renewal, and differentiation of reactive astrocyte-derived neural stem/progenitor cells were described, isolated from the cortical peri-infarct area (Shimada et al., 2012). Following focal cerebral ischemia in rodents, thousands of newborn neuroblasts appear in the striatum over the following weeks and months (Arvidsson et al., 2002; Li and Clevers, 2010; Magnusson et al., 2014), and one-third to two-thirds of newborn neurons are estimated to have been generated by local astrocytes (Magnusson et al., 2014). The expression of miR-181a decreases in neural progenitor cells in the subventricular zone after stroke (Liu et al., 2017), a time of increased neurogenesis, further suggesting that miR-181a impedes neuronal differentiation, a hypothesis supported by our observations of increased MAP2 expression in stem cells treated with miR-181a inhibitor (Fig. 6). Unregulated expression of miR-181a is seen in many malignant cancers (Feng et al., 2018), suggesting some level of control over cell fate. Additionally, MAP2^+^ cells treated with miR-181a inhibitor exhibit altered cell morphology due to the known interactions of the inhibitor with cell fate regulators, but do not stain positive for GFAP, providing additional evidence that they are neurons and not astrocytes. Further studies should reveal whether new neurons born in the presence of miR-181a inhibitor have altered function or assume a normal phenotype when the influence of the inhibitor is removed.

It is also likely that restored neurons in CA1 are derived from another cell type other than astrocytes. Microglia and oligodendrocytes are both prevalent throughout the hippocampus (Zeisel et al., 2015), and have also been demonstrated to have the capacity to dedifferentiate (Grinspan et al., 1996; Yokoyama et al., 2004). Additional experiments should investigate which other cell types in the brain contribute to this phenomenon using genetic fate mapping. Another limitation of the present study is that the analysis of DCX expression and CA1 recovery were performed at 1 week time intervals. Performing the analysis with shorter intervals may reveal a more comprehensive time course of dedifferentiating cells and better define the postinjury therapeutic window for survivors of forebrain ischemia.

In summary, hippocampal CA1 has an endogenous capacity to restore neurons in response to forebrain ischemia. Our observations suggest that this process can be augmented by postinjury treatment with anti-miR-181a. Cellular colocalization and fate mapping further suggest that this process may be mediated by the activation of latent neurogenic pathways in astrocytes. We confirmed *in vitro* that miR-181a inhibition has the capacity to direct stem cells toward a neuronal fate. Future investigations should extend these findings to functional assays to assess the role of miR-181a in cognitive recovery from forebrain ischemia, and in recovery from other models of injury in the adult brain.

## Synthesis

Reviewing Editor: Alfonso Araque, University of Minnesota Decisions are customarily a result of the Reviewing Editor and the peer reviewers coming together and discussing their recommendations until a consensus is reached. When revisions are invited, a fact-based synthesis statement explaining their decision and outlining what is needed to prepare a revision will be listed below. The following reviewer(s) agreed to reveal their identity: Ken Arai, Pedro Tranque. Note: If this manuscript was transferred from JNeurosci and a decision was made to accept the manuscript without peer review, a brief statement to this effect will instead be what is listed below.

Reviewer #1 was satisfied with the response and had no further comments. Reviewer #2 expressed some minor issues that need to be addressed. I concur with reviewer's assessment and I also believe that addressing these minor issues, which seems to be feasibly done in a reasonable short time, will enhance the strength of the manuscript.

I am looking forward to receiving a revised version of the manuscript.

Sincerely,

Alfonso

Specific comments of the reviewers:

Reviewer 1.

No further comments

Reviewer 2.

In this manuscript resubmission, major issues have been appropriately addressed. Only a few minor points need author's attention:

-Line 526: Although B is an enlarged image of A, inexplicably none of the non-specific GFAP labelling observed in the pyramidal layer in A is presented in B

-Line 526: GFAP instead of DCX

-Line 563: New fig 6 legend. Replace “neuronal stem cell” with “neural stem cell” in tittle and text since MAP-2 expressing cells may be both immature astrocytes and neurons. Lack of GFAP expression do not rule out immature astrocytes. Taking cultures to 15DIV or beyond may be the key to get GFAP or Tuj-1 expression in DCX+ cells to clarify the fate of cells induced by miR-181a in future double-staining experiments.
